# Spatial and Temporal Transcriptomic Heredity and Asymmetry in an Artificially Constructed Allotetraploid Wheat (AADD)

**DOI:** 10.3389/fpls.2022.887133

**Published:** 2022-05-16

**Authors:** Xintong Ma, Zhibin Zhang, Guo Li, Xiaowan Gou, Yao Bian, Yue Zhao, Bin Wang, Man Lang, Tianya Wang, Kun Xie, Xiaoming Liu, Bao Liu, Lei Gong

**Affiliations:** ^1^Key Laboratory of Molecular Epigenetics of the Ministry of Education (MOE), Northeast Normal University, Changchun, China; ^2^School of Life Sciences, Jiangsu Normal University, Xuzhou, China; ^3^School of Life Sciences, Liaoning Normal University, Dalian, China; ^4^Jia Sixie College of Agriculture, Weifang University of Science and Technology, Shouguang, China

**Keywords:** allopolyploid wheat, *Triticum urartu*, *Aegilops tauschii*, differentially expressed genes, homoeolog expression bias, WGCNA

## Abstract

Polyploidy, or whole-genome duplication (WGD), often induces dramatic changes in gene expression due to “transcriptome shock. ” However, questions remain about how allopolyploidy (the merging of multiple nuclear genomes in the same nucleus) affects gene expression within and across multiple tissues and developmental stages during the initial foundation of allopolyploid plants. Here, we systematically investigated the immediate effect of allopolyploidy on gene expression variation in an artificial allopolyploidy system consisting of a constructed allotetraploid wheat (AADD genome, accession AT2) and its diploid progenitors *Triticum urartu* and *Aegilops tauschii*. We performed comprehensive RNA sequencing of 81 samples from different genotypes, tissues, and developmental stages. First, we found that intrinsic interspecific differences between the diploid parents played a major role in establishing the expression architecture of the allopolyploid. Nonetheless, allopolyploidy *per se* also induced dramatic and asymmetric patterns of differential gene expression between the subgenomes, and genes from the D subgenome exhibited a more drastic response. Second, analysis of homoeolog expression bias (HEB) revealed that the D subgenome exhibited significant expression bias and that *de novo*-generated HEB was attributed mainly to asymmetrical differential gene expression. Homoeolog-specific expression (HSE) analyses showed that the *cis*-only regulatory pattern was predominant in AT2, reflecting significant divergence between the parents. Co-expression network analysis revealed that homoeolog expression connectivity (HEC) was significantly correlated with sequence divergence in *cis* elements between subgenomes. Interestingly, allopolyploidy-induced reconstruction of network modules was also associated with different HSE patterns. Finally, a transcriptome atlas of spike development demonstrated that the phenotypic similarity of AT2 to *T. urartu* may be attributed to the combination of relatively stable expression of A-subgenome genes and drastic downregulation of their D-subgenome homoeologs. These findings provide a broad, multidimensional characterization of allopolyploidy-induced transcriptomic responses and suggest that allopolyploidy can have immediate and complex regulatory effects on the expression of nuclear genes.

## Introduction

As an important mechanism of angiosperm speciation, polyploidy (whole genome duplication, WGD) has far-reaching consequences for plant evolution and ecology (Chen, 2007; Yoo and Wendel, 2014; Soltis et al., 2015; Van de Peer et al., 2017; Wong et al., 2020). All angiosperm species have undergone one or more ancient WGD events during their evolutionary history and are, therefore, paleopolyploids (Jiao et al., 2011). Hybridization of divergent species followed by genome doubling produces allopolyploids, which often benefit from genetic and functional innovations and show increased adaptive potential compared with their diploid ancestors (Chen, 2007; Li et al., 2014; Zhang et al., 2020). In allopolyploids, merging and doubling of divergent genomes in the nucleus may have joint effects on global gene expression patterns, giving rise to differentially expressed genes (DEGs), homoeolog expression bias (HEB), and expression level dominance (ELD), which have, together, been termed “transcriptome shock” (Buggs et al., 2011). These transcriptomic changes may underlie phenotypic and adaptive variation in the early stage of polyploid formation (Doyle et al., 2008; Jackson and Chen, 2010).

HEB is defined as the preferential expression of one homoeolog relative to the other and occurs frequently with varying levels of magnitude (balanced or unbalanced) in both natural and synthetic allopolyploids (Feldman and Levy, 2009; Grover et al., 2012; Yoo et al., 2014; Wang et al., 2016; Wu et al., 2018), including those of *Arabidopsis* (Chang et al., 2010), cotton (Yoo et al., 2013), oilseed rape (Wu et al., 2018; Li et al., 2020), and wheat (Pfeifer et al., 2014; Ramírez-González et al., 2018). There are two major patterns of HEB; parent-legacy HEB and *de novo*-generated HEB (Buggs et al., 2014). The former is due to intrinsic *cis*-element-related expression divergence between orthologs in the different parents, whereas the latter results from allopolyploidy-induced differential gene expression and represents a key aspect of homoeolog expression reprogramming. HEB can eventually lead to subgenome dominance at the expression level, subgenome-preferential inheritance, and variations in phenotype and stress tolerance (Feldman et al., 2012; Powell et al., 2017; Van de Peer et al., 2017).

Allopolyploidy entails the merging of homeologous regulatory variation and may, therefore, lead to differences in gene expression through interacting *cis-* and *trans-*regulatory factors (Chaudhary et al., 2009). Homoeolog-specific expression (HSE) analysis, which considers homoeolog expression divergence relative to expression divergence between parental orthologs, has been used to investigate the relative contributions of *cis-* and *trans-*acting regulatory changes after allopolyploidy (Wittkopp et al., 2004). HSE analysis can help to distinguish the roles of novel subgenome interactions from those of progenitor regulatory interactions (Song et al., 2019). Recent studies of *cis* and *trans*regulation in resynthesized and natural allotetraploid *Arabidopsis* (Shi et al., 2012) and in wild and domesticated allotetraploid cotton (Bao et al., 2019) have revealed how *cis* and *trans* regulation affect gene expression divergence during the evolution and domestication of allopolyploids. However, previous studies have focused on only one or a few tissues at specific developmental stages (Shi et al., 2012; Bao et al., 2019; Hu and Wendel, 2019); general or dynamic characteristics of the regulation of HEB and HSE across multiple developmental stages of different allopolyploid tissues remain largely uncharacterized.

Weighted gene co-expression network analysis (WGCNA) is commonlyused to construct gene co-expression networks, associate modules with key genes that control phenotypes of interest, and characterize gene expression dynamics in multiple tissues and/or developmental stages (Umer et al., 2020). Its underlying hypothesis is that genes grouped into shared modules harbor close biological relatedness and/or exhibit similar patterns of regulation. A number of studies have used WGCNA to identify gene co-expression networks in polyploid crops (Pfeifer et al., 2014; Hu et al., 2016; Li et al., 2016), which are thought to exhibit regulatory novelty through modifications of duplicated gene co-expression networks (Gallagher et al., 2016; Takahagi et al., 2018). However, more comprehensive studies of novel co-expression networks in specific allopolyploids are needed to determine whether allopolyploidy can reconstruct networks of co-expressed homoeologs within and across modules and to investigate how this is associated with altered HSE patterns.

The *Triticum/Aegilops* species complex has long been regarded as an interesting model to investigate mechanisms of species diversification caused by hybridization and allopolyploidization (Matsuoka, 2011). The *Triticum* and *Aegilops* genera referred to as the wheat group include 13 diploids and 18 allopolyploids, which belong to eight distinct but related genome groups (A, D, S, M, U, C, N, and T) (Feldman and Levy, 2015). Most allopolyploids in the wheat group are presumed to share a common unaltered (pivotal) subgenome (U, D, or A), together with one or two modified (differential) subgenomes in a model referred to as “pivotal-differential” genome evolution (Mirzaghaderi and Mason, 2017). Both the A and D subgenomes are pivotal genomes in terms of different aspects: the A subgenome controls morphological and reproductive traits, such as the inflorescence morphology of tetraploid wheat (BBAA) (Feldman et al., 2012; Wang et al., 2016). It has also been reported that the A subgenome is dominant over the B subgenome in terms of genomic stability in tetraploid wheat (Pont et al., 2013). The D subgenome in allohexaploid wheat mainly controls responses to biotic and abiotic stresses and regulates adaption to ecological conditions (Feldman and Levy, 2009). Motivated by these observations, we proposed an intriguing question: what would be the outcome if the two pivotal A and D genomes were merged by allopolyploidization? Because there is no allopolyploid wheat composed of the A and D subgenomes in nature, we constructed an ideal synthetic AADD allotetraploid wheat lineage in an earlier study (Gou et al., 2018). Based on this artificially synthesized allotetraploid wheat, the chromosomal outcome of merging and doubling the A and D subgenomes was initially characterized (Zhang et al., 2020). We detected numerical chromosomal variation that exhibited significant subgenome bias (higher aptitude for A-subgenome chromosome gain and D-subgenome loss). Moreover, seed setting and spike density scaled with an increase in A-subgenome chromosomes and a decrease in D-subgenome chromosomes (Gou et al., 2018). However, the homoeolog expression patterns of this AADD allopolyploid lineage remain to be explored both in terms of HEB and HSE and in terms of their dynamics across multiple tissues and developmental stages.

In this study, we analyzed RNA-seq data from nine tissues at different developmental stages in the previously constructed synthetic allotetraploid wheat lineage (AADD, AT2) and its diploid parents *Triticum urartu* (AA) and *Aegilops tauschii* (DD). We systematically investigated allopolyploidy-induced variations in gene expression and crosstalk in expression regulation between the subgenomes. Global changes in gene expression were characterized by comparing the transcriptomes of AT2 with those of its parents. Based upon comparative analysis of HEB patterns for homoeolog pairs, we investigated whether and how transcriptome asymmetry was established after the doubling and merging of the two pivotal genomes. Furthermore, *cis-* and *trans-*regulatory HSE variants accompanying wheat allopolyploidization were characterized and integrated through the construction of co-expressed homoeolog networks. Finally, the potential relationship between overall A-subgenome dominance and the similarity in inflorescence morphology between AT2 and its diploid AA parent was investigated throughout the course of inflorescence development.

## Results

### Karyotype Characteristics and Transcriptome Profiling of AADD and Its Diploid Parents Across Developmental Stages

**Figure 1:**
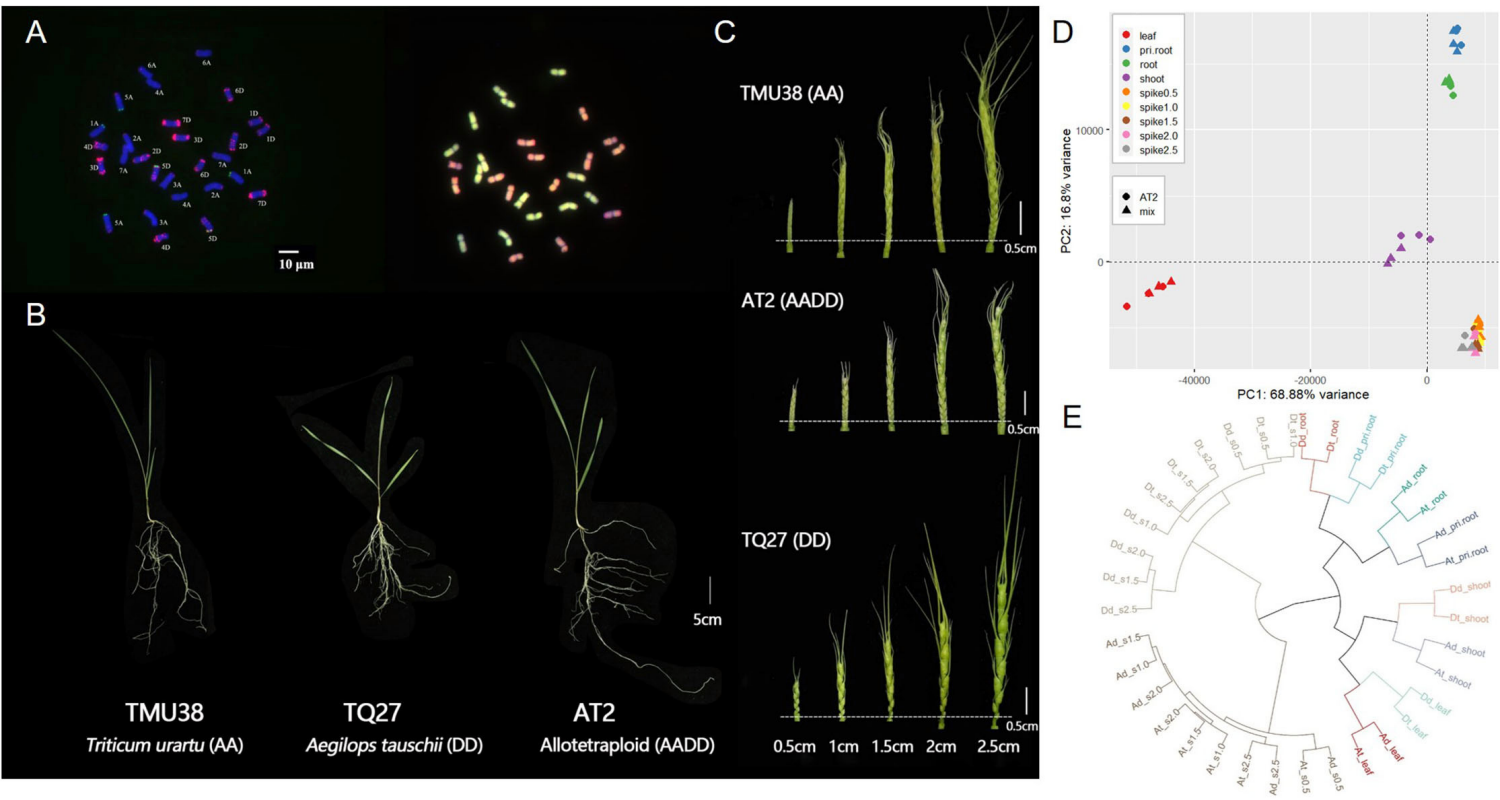
Karyotypes, phenotypes, and spatio-temporal gene expression clustering in synthetic allotetraploid wheat (AT2, AADD) and corresponding parental species (TMU38 and TQ27 for AA and DD, respectively). **(A)** Euploid karyotypes of AT2 detected using fluorescent *in situ* hybridization (FISH, left) and genomic *in situ* hybridization (GISH, right). The DNA probes of FISH analysis are pSc119.2 (green) and pAS1 (red); **(B)** The phenotype of trefoil stage seedling of AT2 and parent species; **(C)** The spike morphology of AT2 and parental diploids from 0.5 to 2.5 cm in length; **(D)** Principal component analysis (PCA) of transcriptomic expression profiles in AT2 and parental diploids (represented by “Mix”; see Materials and Methods). Colors and shapes denote the different tissues/stages and genotypes, respectively; **(E)** Hierarchical clustering of homoeolog expression in AT2 and parents. Ad and Dd denote the ortholog pairs in AA and DD diploid parents; At and Dt denote the homoeolog pairs in AT2. The bar of karyotype and phenotype photos is shown as white lines.

We produced a nascent synthetic allotetraploid wheat (AADD) through hybridization and artificial WGD of two diploid parental species of bread wheat, *Triticum urartu* (AA), and *Aegilops tauschii* (DD). To avoid previously reported noise effects caused by dramatic changes in chromosome number and structure in AT2 (Zhang et al., 2013; Gou et al., 2018), all individuals of AADD wheat (accession AT2) were initially karyotyped. Karyotyping was performed by sequential FISH with two DNA probes (pSc119.2, green; pAS1, red) followed by GISH, which enabled the identification of all A- and D-subgenome homoeologous chromosome groups in AT2 individuals (Figure 1A). Individuals without variations in chromosome number and structure (euploids, 2n = 28) were used for subsequent tissue collection and RNA-seq analyses.

We sampled various tissues from AT2 and the diploid AA and DD parents at three developmental stages: 4 days after germination (primary roots and shoots), the trefoil stage (roots and leaves), and the inflorescence stage (inflorescences, 0.5–2.5 cm in length) (Figures 1A,C, Materials and Methods). To obtain a global transcriptomic atlas of all tissues, developmental stages, and genotypes, we performed RNA-seq analysis of 81 samples (three genotypes × nine tissues/stages × three replicates). In total, we generated ~390 Gb of clean data, with an average of 22.9 million reads covering each subgenome (Supplementary Table S1). An *in silico* parental mix (hereafter denoted Mix) was constructed by mixing the diploid parental RNA-seq data at a ratio of 1:1 to represent for the orthologous gene expression divergence in those two parents. After aligning the sequencing reads and retaining only genes with >1 TPM (transcripts per million reads) in at least one sample, we obtained an expression matrix of 50,194 genes in AT2 and Mix for use in principal component analysis (PCA) to generate an overview of gene expression in the AADD synthetic allotetraploid lineage. The first two principal components (PC1 and PC2) explained 85.76% of the total variance and grouped all samples into four distinct clusters according to tissue type (leaf, shoot, two root tissues, and five inflorescence tissues). Tissue type, therefore, appeared to be the main factor responsible for differences in gene expression between samples. This was consistent with the grouping pattern reported in other studies (Appels et al., 2018) (Figure 1D). To further examine the transcriptome atlas between homoeologous subgenomes, 9,642 homoeologs from the A and D subgenomes expressed in at least one tissue were used to perform hierarchical clustering analysis. Consistent with the PCA results, hierarchical clusters primarily reflected tissue types (Figure 1E). However, within each tissue cluster, subgenomes in the allopolyploid and corresponding parental genomes tended to form sub-clusters (Figure 1E), indicating that interspecific differences and parental legacy were the most important factors that influenced global transcription patterns in the synthetic allopolyploid lineage.

### Allopolyploidization Induced Dramatic and Asymmetric Differences in Expression Between the Subgenomes

**Figure 2:**
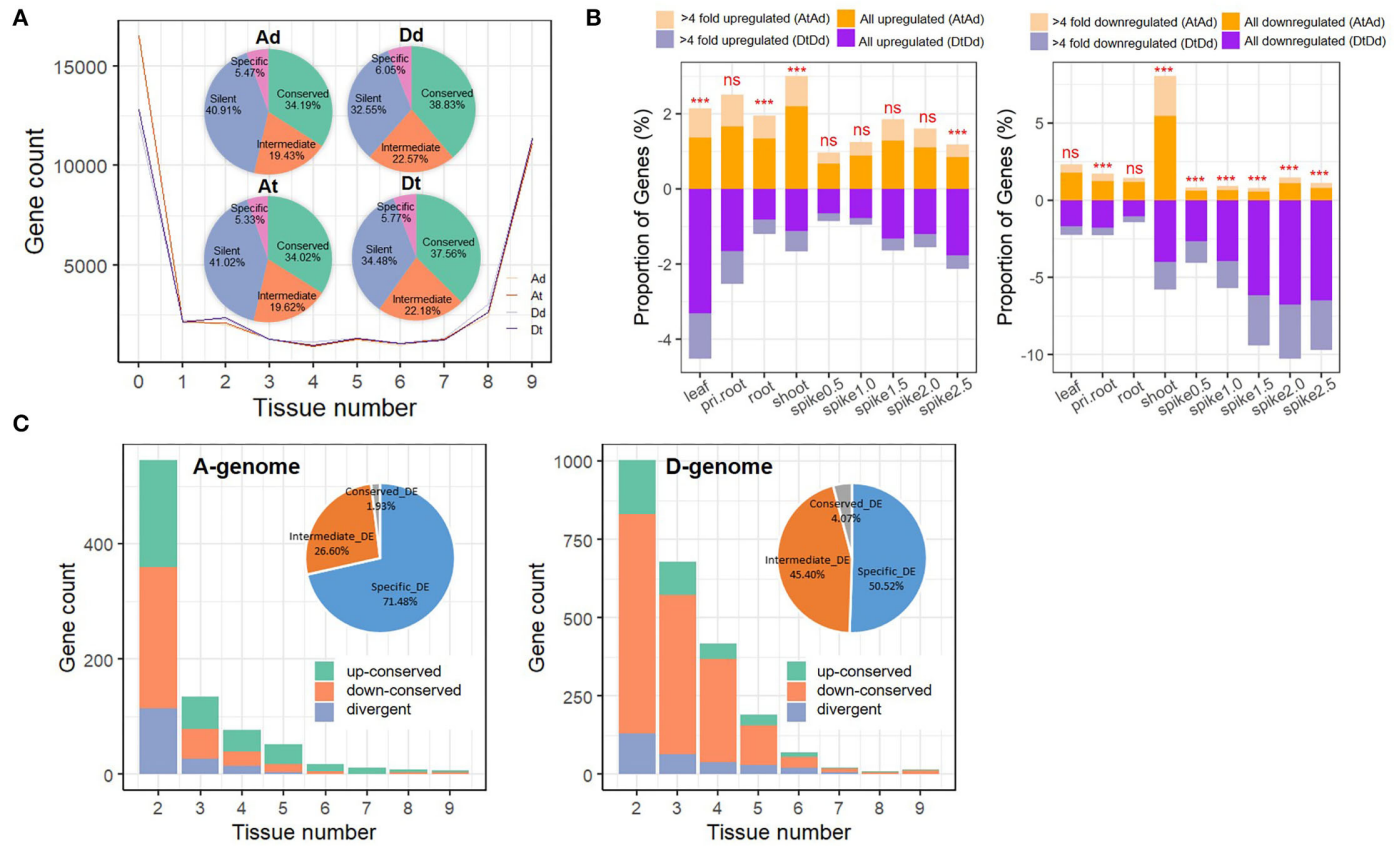
Asymmetric expression response of subgenomes to allopolyploidy in AT2. **(A)** Gene expression patterns among nine tissues/stages in AT2 and parents. Gene expression distributions of different tissues/stages among genomes/subgenomes are shown in the line chart. The numbers of genes that marked as “silent” (without expression in any tissue; 0 tissue), “specific” (TPM > 1 in one tissue), “intermediate” (TPM > 1 in 2–7 tissues) and “conserved” (TPM > 1 in 8–9 tissues) among genomes/subgenomes are specified in the pie chart; **(B)** The proportion of up/downregulated genes (At *vs*. Ad and Dt *vs*.Dd) in different tissues/stages. Respective statistical significance of difference in the number of DEGs between A and D subgenomes is specified above each column (***denotes significance at *p*-value < 0.001; ns denotes “no significance”); **(C)** Conservation of DEGs among tissues/stages in A (the left panel) and D (the right panel) subgenomes. The number of three gene groups genes, including “up-conserved” (genes in all given tissue numbers are upregulated, green color) and “down-conserved” (genes in all given tissue numbers are downregulated, orange color) and “divergent” (genes in some tissues are downregulated but upregulated in other tissues, blue color) groups, are shown in barplot. DEGs occurred in only one tissue (specific_DE, blue color), 2–7 tissues (intermediate_DE, orange color) and 8–9 tissues (conserved_DE, gray color) are presented in the pie chart.

To summarize and compare differences in transcriptomic expression between the parental diploid genomes and their respective allopolyploid subgenomes, we categorized parental diploid orthologs (Ad and Dd) and allopolyploid homoeologs (At and Dt) in terms of their expression conservation across tissues and developmental stages. In brief, genes were categorized based on the ubiquity of their expression: genes expressed in all or most tissues (8–9 tissues/stages) were placed in the “conserved” group, genes with intermediate expression (2–7 tissues/stages) in the “intermediate” group, genes expressed in a single tissue in the “specific” group, and genes with no expression in the “silent” group (Figure 2A). Intriguingly, most orthologs and homoeologs in both the parental diploids and the allotetraploids consistently displayed either a “conserved” or a “silent” expression pattern (34.02–38.83% and 32.55–41.02%, respectively; Figure 2A), giving rise to a bimodal pattern for the number of tissues in which genes were expressed. The “intermediate” and “specific” groups contained 19.43–22.57% and ~6% of all homoeologs (Figure 2A). Major differences in “conserved,” “intermediate,” and “silent” gene numbers between the allotetraploid At and Dt subgenomes were mainly inherited from their diploid parents (Ad *vs*. Dd; Figure 2A). We found majority of genes (91.21 and 89.63% in A and D subgenomes, respectively) inherited parental types in AT2. Specifically, in A genome, the “intermediate” group showed maximum change (2.86%), whereas the “conserved” group showed minimum change (1.3%); similar to A genome, the “intermediate” group showed maximum change (3.73%), whereas the “silent” group showed minimum change (1.38%) in D genome (Supplementary Figure S1). Such a result suggests that interspecific differences (*cis* effects) rather than allopolyploidy itself have an important role in regulating transcriptome differences between the two allopolyploid subgenomes. Furthermore, when we compared the expression level group compositions between At and Ad and between Dt and Dd, the At and Ad genomes exhibited almost identical expression patterns (χ^2^-test, *p*-value = 0.45; Figure 2A; Supplementary Figure S2); in contrast, the Dt subgenome exhibited greater differences in gene activation and silencing relative to the Dd genome at the whole-tissue scale after allopolyploidization (χ^2^-test, *p*-value < 0.001; Figure 2A; Supplementary Figure S2).

Accordingly, we focused on characterizing expression differences in gene pairs from each homoeologous subgenome *vs*. its parental diploid genome (At *vs*. Ad and Dt *vs*. Dd, abbreviated as AtAd and DtDd hereafter). We identified 3,474 (13.44%) and 5,305 (19.41%) differentially expressed genes (DEGs) (>2-fold changes) in at least one tissue between two A and D genomes, respectively. Specifically, in the pair of At *vs*. Ad (AtAd), there were 135–455 (0.67–2.2%) upregulated genes and 115–1,133 (0.55–5.47%) downregulated DEGs in At; in the pair of Dt *vs*. Dd (DtDd), there were 138–624 (0.65–3.31%) upregulated genes and 229–1,548 (1.03–6.77%) downregulated genes in Dt (Figure 2B). In particular, the proportion of downregulated DEGs in spike tissues (from 0.5 to 2.5 cm) was much higher in the DtDd comparison than in the AtAd comparison (Fisher's exact test, *p*-value < 0.001), and this proportion increased gradually with spike development (from 2.65% in 0.5-cm spikes to 6.49% in 2.5-cm spikes, Figure 2B). We next focused on genes that showed the greatest response to allopolyploidization (>4-fold changes). The expression trends observed across all DEGs in paired comparisons were also observed in this group of allopolyploidy-responsive genes (Figure 2B). GO analysis of DEGs identified in DtDd comparison across tissues showed that the DEGs downregulated in spike tissues were enriched in biological process terms related to photosynthesis, the cellulose catabolic process, and the activities of various enzymes (Supplementary Figure S3). The larger percentage of D subgenome genes downregulated in spike tissues may explain, at least in part, why the spike phenotype of AT2 is more similar to that of *T. urartu* (Figure 1C).

The DEGs identified in paired comparisons (AtAd and DtDd) were also categorized into groups based on the number of tissues in which they were expressed, as described above. We found that 71.48% (1,967) of all DEGs in the AtAd comparison were tissue specific (expressed in a single tissue), and the remaining 28.53% (785) were shared among at least two tissues; these included 26.6% (732) intermediate DEGs (2–7 tissues) and 1.93% (53) conserved DEGs (8–9 tissues) (Figure 2C). The DEG categorization differed markedly in the DtDd comparison: The proportion of tissue-specific DEGs decreased to 50.52% (2,319), and the proportions of intermediate and conserved DEGs increased to 45.4% (2,084) and 4.07% (187), respectively (Figure 2C). To investigate whether the same gene has different expression response (up- or downregulated) to allopolyploidy among different tissues, the gene, which is upregulated (down-regulated) in all checked tissues, was defined as “up-conserved” (“down-conserved”), whereas that with upregulated in some tissues but downregulated in other tissues was defined as “divergent.” For the DEGs expressed in at least two tissues, consistent up- or downregulation in the AtAd (79.95%, 682) and DtDd (88.14%, 2,119) comparisons implied that these genes showed a convergent response to allopolyploidization (in all tissues where they were expressed). The proportion of such DEGs increased gradually as the number of tissues increased in the AtAd comparison but showed little change with tissue number in the DtDd comparison (Figure 2C). The specific up- and downregulation pattern differed between the AtAd and DtDd comparisons. Specifically, there were relatively similar proportions of up- and downregulated DEGs in the AtAd comparison (40.68 *vs*. 40.56%, χ^2^-test, *p*-value = 0.9697), but there were far more downregulated DEGs in the DtDd comparison (16.1% up *vs*. 72.05% down, χ^2^-test, *p*-value < 0.001). A sizable fraction of the DEGs, 18.76% (AA) and 11.86% (DD), showed divergent responses to allopolyploidization; in other words, these DEGs showed a different direction of regulation in at least two tissues, unlike the DEGs that showed a convergent response (Figure 2C). Together, these results show that allopolyploidy can lead to expression differences in given tissues for both subgenomes, but the Dt genome showed greater expression differences than the At genome in response to allotetraploidy.

### Homoeolog Expression Bias Establishes Transcriptional Subgenome Dominance in Response to AADD Allopolyploidy

One of the most general phenomena induced by allopolyploidization is subgenome dominance, which is established by homoeolog expression bias (Grover et al., 2012). Accordingly, we identified 9,642 homoeologous and orthologous gene pairs in AADD and its diploid parents and explored transcriptional subgenome dominance in all allopolyploid samples. To dissect and trace the underlying HEB variation in response to allopolyploidy, we classified homoeolog pairs into four major groups according to their HEB status in the parental mix and AT2 (Materials and Methods; Supplementary Table S3). We found that the majority of homoeolog pairs inherited the parental HEB pattern across tissues (Group *i*, 90.87–94%), and the proportion of A-biased homoeologs was significantly lower than that of D-biased homoeologs (6.82–8.84% in A subgenome *vs*. 13.66–14.92% in D subgenome; Fisher's exact test, *p*-value < 0.001), suggesting that parental legacy is a major determining factor in the transmission of gene expression patterns. Homoeolog pairs that had lost parental expression bias (Group *ii*, 2.13–5.73%) and pairs with *de novo* expression bias (Group *iii*, 2.71–4.02%) were moderately common (Supplementary Table S3). In Group *iii*, there was a more pronounced *de novo* expression bias toward the A subgenome (1.39–2.49% for A-HEB, 1.22–1.68% for D-HEB; Supplementary Table S3). Homoeologs with reversed HEB (Group *iv*) were relatively uncommon and were scattered among different tissues and stages.

**Figure 3:**
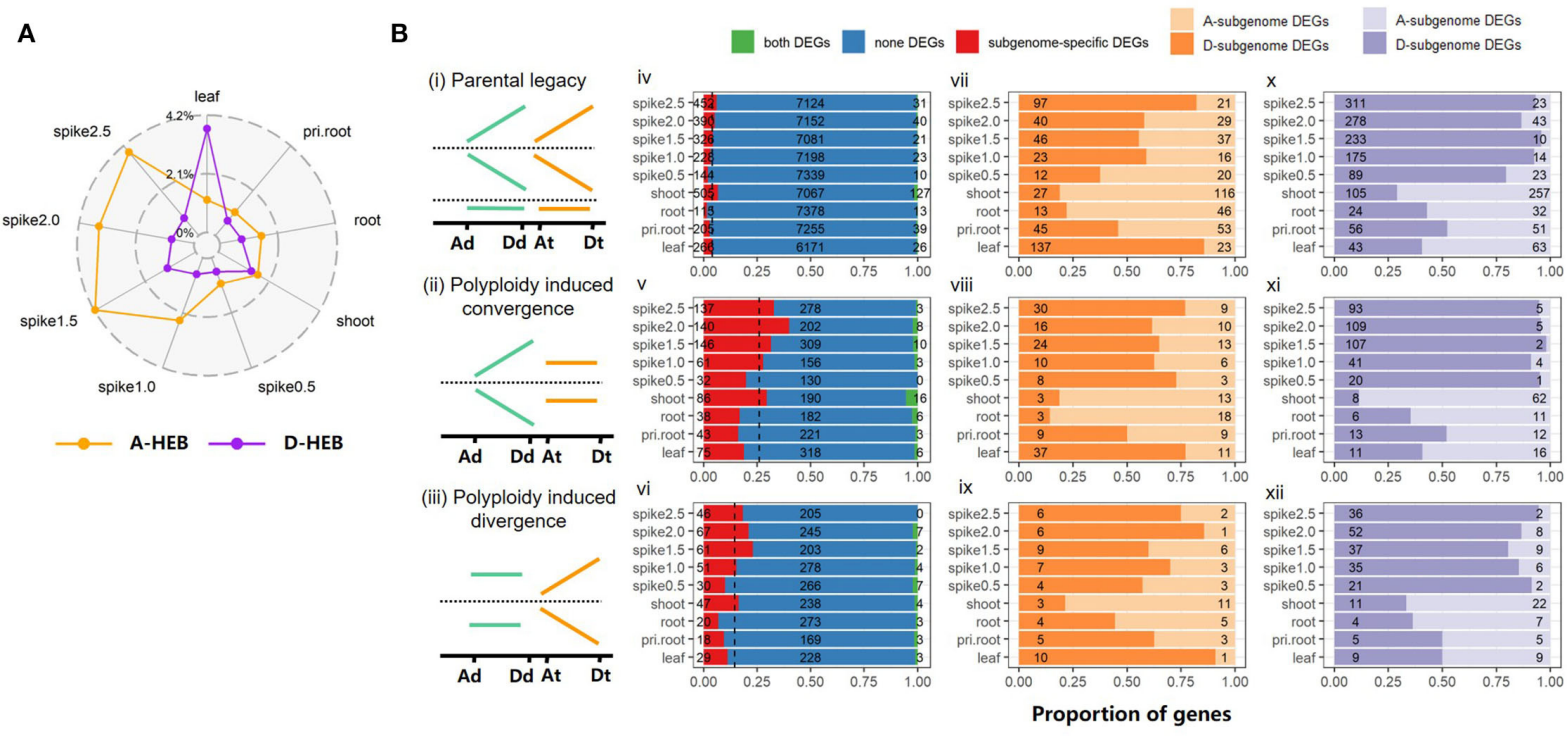
Homoeolog expression bias (HEB) and the contribution of DEG to HEB. **(A)** The proportion of HEB genes across tissues/stages in AT2. Orange and purple colors represent the A-HEB and D-HEB genes, respectively; **(B)** Contribution of DEGs on different homoeolog classes. Schematic illustration of three homoeolog categories, including (i) parental legacy, (ii) allopolyploidy-induced convergence and (iii) allopolyploidy-induced divergence, are presented in the first column. The relative expression levels of orthologs/homoeologs in different genomes/subgenomes are indicated by relative height of lines. For each category among tissues, the number of three types of homoeolog pairs, including both DEGs (all two mates of a homoeolog pair are DEGs, green), subgenome-specific DEGs (only one mate of a homoeolog pair is DEG, red), and none-DEGs (all two mates of a homoeolog pair are not DEGs, blue), are shown in the second column (iv–vi); A- (light orange color) and D-upregulated (dark orange color) DEGs (vii–ix), as well as A- (light purple color) and D-downregulated (dark purple color) DEGs (x–xii) are summarized in the third and fourth columns, respectively.

Based onthe HEB patterns in the parents and AT2, we calculated the net HEB characterization by comparing the expression of each homoeolog to that of its parental orthologs (Materials and Methods). As shown in Figure 3A, more genes exhibited D-subgenome dominance than A-subgenome dominance in leaf tissues (3.72% D-HEB *vs*. 1.16% A-HEB). However, other tissues, especially the five spike tissues, showed more A-HEB: 0.99–4.17% A-HEB *vs*. 0.53–1.17% D-HEB (Figure 3A; χ^2^-test, *p*-value < 0.001).

To explore the contribution of allopolyploidy-induced differential gene expression to HEB variation, we divided each of the HEB groups above (except Group *iv*) into three classes based on whether both, neither, or one of the homoeologs from the A and D subgenomes was DEG (Figure 3B). The latter group of subgenome-specific DEGs was subdivided into A-subgenome DEGs and D-subgenome DEGs. The proportion of subgenome-specific DEGs was lower in Group *i* (from 1.53% in root tissue to 6.56% in shoot tissue) than in Group *ii* (from 16.1% in primary root tissue to 40% in 2.-cm spike tissue) and Group *iii* (from 6.76% in root tissue to 22.93% in 1.5-cm spike tissue), which probably contributed to the significant difference in HEB (Figure 3B; χ^2^-test, *p*-value < 0.001). In addition, there were more downregulated homoeologs than upregulated homoeologs in all three groups (Student's *T*-test, *p*-value = 0.02 for Group *i, p*-value = 0.04 for Group *ii*, and *p*-value = 0.01 for Group *iii*). In particular, there were more downregulated D-subgenome homoeologs than A-subgenome homoeologs at all five spike developmental stages (29.01–95.88% *vs*. 4.12–70.99% in Group *i*, 11.43–98.17% *vs*. 1.83–88.57% in Group *ii*, and 33.33–94.74% *vs*. 5.26–66.67% in Group *iii*), suggesting that the overall A-subgenome dominance in AT2 is correlated with dramatic downregulation of gene expression in the Dsubgenome (Figure 3B).

To measure the expression correlation between homoeologs at the network level, we also constructed a homoeolog co-expression network. As shown in Supplementary Figure S4, the homoeologs were divided into three distinct groups (A–C) based on their different expression connectivity scores (abbreviated HEC hereafter) among all tested tissues. The higher the connectivity, the more the homoeologs are assumed to share the same expression profile, whereas lower connectivity indicated greater expression divergence (Materials and Methods). In line with the observed increase in expression divergence, the levels of non-synonymous (Ka) and synonymous substitutions (Ks) and nucleotide divergence in homoeolog promoters gradually increased in the three groups as expression connectivity decreased (leveraged expression divergence) in both Mix and AT2 (Supplementary Figure S4A, ANOVA and Tukey's HSD test, *p*-value < 0.05). This result indicates that variation in *cis*-elements and/or amino acid sequences (i.e., *cis* effects) was likely to underlie the expression divergence between homoeologs.

By characterizing changes in HEC induced by allopolyploidization, we found that most homoeologs inherited the parental expression pattern (55.85%, 28.29%, and 3.94% for the three groups) and maintained relative high HEC scores (84.14% with scores >0.3, Group A + B), implying that the function and expression of most homoeologs remained similar before and after allopolyploidy. For homoeologs that switched between groups, allopolyploidization tended to increase rather than reduce expression relatedness between homoeologs (8.39% from low HEC to high HEC vs. 3.54% from high HEC to low HEC, χ^2^-test, *p*-value < 0.001), which implies potential cross-talk between subgenomes *via trans* factors in the allopolyploid (Supplementary Figure S4B).

### HEC Changes Induced by Allopolyploidy in AT2 Are Associated With Homoeolog-Specific Expression

**Figure 4:**
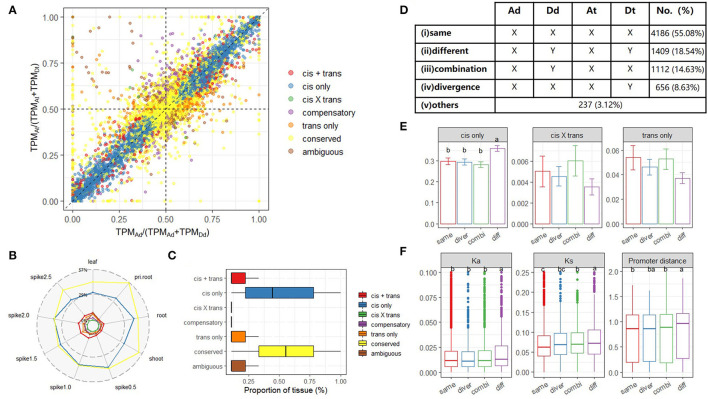
Homoeolog-specific expression (HSE) analysis across tissues/stages. **(A)** A scatterplot shows the relationship between the expression ratio of A allele at a diploid level [*x*-axis, TPM_Ad_/(TPM_Ad_+TPM_Dd_)] and that of A homoeolog at a tetraploid level [*y*-axis, TPM_At_/(TPM_At_+TPM_Dt_)] for genes in root tissue. The colors represent different HSE patterns determined for each gene; **(B)** Proportion of homoeolog pairs classified into each HSEpattern; **(C)** The conservation degree of different HSE patterns among tissues; **(D)** The number of homoeolog pairs among five co-expression groups of quadruplets (orthologs Ad and Dd as well as homoeologs At and Dt) in AT2 based on the co-expression network. For a given module, “X” means a gene is belonged to this module, whereas “Y” means a gene is not in such a module; **(E)** The proportion of different types of HSE genes in four co-expression groups. Error bars indicate the variance among nine tissues. One-way ANOVA and multiple comparisons were performed to compare the ratio among co-expression groups (*p*-value < 0.05); **(F)** Evolutionary features of homoeologs belonged to different co-expression groups are denoted in each sub-panel, including Ka (non-synonymous mutation), Ks (synonymous mutation), and sequence divergence of the promoter region. One-way ANOVA is used to compare the difference in each evolution feature among co-expression groups (*p*-value < 0.05).

To explore *cis*-*trans* regulatory mechanisms at the initial stage of allopolyploidy, we characterized homoeolog-specific expression (abbreviated as HSE hereafter) for 9,642 expressed homoeologs in all tested tissues. Based on a published classification method (Bao et al., 2019), we identified seven categories: conserved, *cis*-only, *trans*-only, *cis* + *trans, cis* × *trans*, compensatory, and ambiguous (Materials and Methods). An example summary of gene homoeologs in these categories from root tissue is shown in Figure 4A. The overall numbers of genes in a given category were relatively similar in different tissues and stages (Supplementary Figure S5). Notably, with the exception of the conserved category, *cis*-only homoeologs were most common in all tested tissues (average, 36.59%, Figure 4B), whereas *cis* × *trans* homoeologs were least common (average, 0.36%, Figure 4B). When we focused on the frequency of each category, *cis*-only homoeologs were present in the highest proportion of tissues, but the median proportion for *cis*-only homoeologs was still <50% (0.44). All these results imply that HSE regulation among different tissues and stages of synthetic AT2 wheat was extremely variable and complex (Figure 4C).

To further explore allopolyploid-induced variations in HEC, we tested whether or not homoeologs had undergone a transition between different modules based on the homoeolog co-expression network described above. For each quadruplet (Ad–Dd ortholog pairs in diploid parents and At–Dt homoeolog pairs in AT2), we defined different rewiring groups based on whether the four members belonged to the same or different co-expression modules. We classified the 9,642 quadruplets into five distinct groups: (*i*) conserved same: Ad, Dd, At, and Dt belonged to the same module; (*ii*) conserved different: Ad and Dd werein different modules, but Ad and At were in the same module, as were Dd and Dt; (*iii*) convergence: Ad and Dd were in different modules, but At and Dt were in the same module; *(iv*) divergence: Ad and Dd were in the same module, whereas At and Dt were in different modules, and (*v*) others: any pattern that did not match one of modules *i*–*iv* (Figure 4D).

As expected, the majority of quadruplets (4,186, 55.08%) showed a conserved expression pattern in which all four members belonged to the same co-expression module (Group *i*). Group *ii* contained 1,409 (18.54%) quadruplets, and we inferred that such a parent-mimic pattern (in which the subgenomes differed and matched their respective parents) may reflect divergence of *cis* elements in the parents after species differentiation. Indeed, when HSE patterns were considered, Group *ii* possessed the highest proportion of *cis*-only homoeologs (Figure 4E; Games-Howell *post-hoc* test, *p*-value < 0.001). Furthermore, Group *ii* had the highest Ka and Ks values and nucleotide diversity between homoeologous promoters, providing further evidence for the role of *cis* regulation (Figure 4F; Games-Howell *post-hoc* test, *p*-value < 0.05). Notably, the number of quadruplets for which only the parent orthologs were in different modules (Group *iii*; 1,112, 14.63%) was significantly higher than the number for which only the tetraploid homoeologs were in different modules (Group *iv*; 656, 8.63%) (Figure 4D; χ^2^-test, *p*-value < 0.001). The former result may be related to the allopolyploid environment with a shared *trans* regulator, whereas the latter appears to be associated with subgenome dominance (asymmetry).

### Gene Expression Variation During Spike Development in AT2

A notable phenotypic feature of AT2 is its general morphological mimicry of *T. urartu* rather than *Ae. tauschii* during spike development, especially for the spikelet shape and awn length: Relative to *T. urartu* and AT2 having thin and long spikelet, the spikelet of *Ae. tauschii* is wider and shorter (Figure 1C). We hypothesized that spike development-related genes (SDRGs) that were downregulated in the D subgenome and/or dominant in the A subgenome may have participated, independently or together, in the regulation of spike development after AADD allopolyploidy.

**Figure 5:**
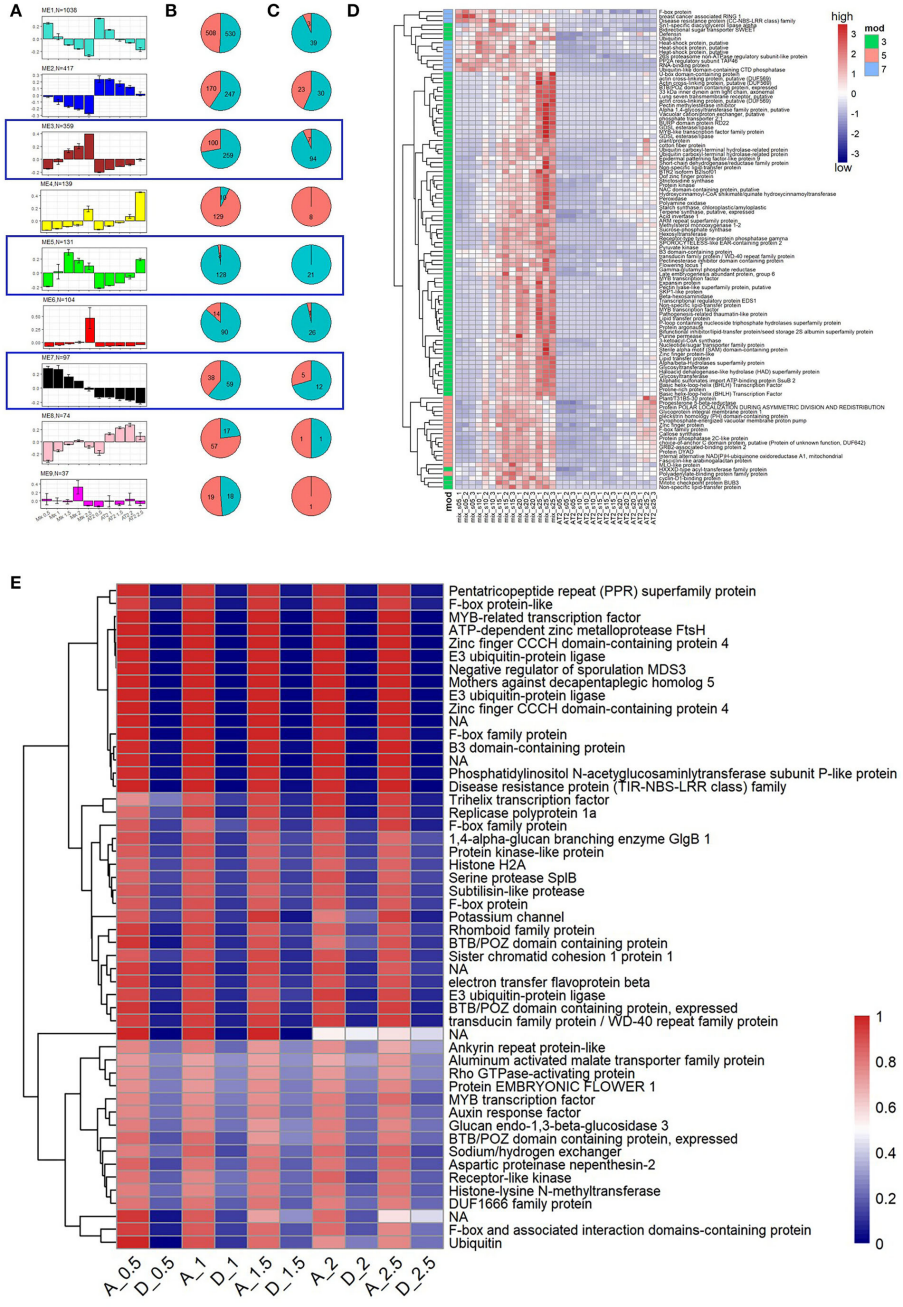
Gene expression patterns during spike development in AT2 and parents. **(A)** SDRGs in modules of co-expression network. Bar plots present eigengene values centered by mean across developmental stages. Error bars represent the standard errors among three biological replicates for each spike developmental stage. Module color and number of module member genes are noted above each bar graph; **(B,C)** pie charts summarize the SDRG ratios of A (red color) and D subgenomes (blue color) for all the genes and the differentially expressed SDRGs in each module, respectively. The blue box indicates that the module contains stably downregulated SDRGs; **(D)** Expression levels (measured as Z-transformed TPM value) of stably downregulated SDRGs in foregoing three co-expression modules; **(E)** The expression ratio of SDRGs bias toward the A subgenome [TPM_At_/(TPM_At_ + TPM_Dt_)]. Gene functions are listed on the right side of the heatmaps.

To explore this hypothesis, we constructed a new gene co-expression network of five spike developmental stages based on the expression of 2,561 SDRGs (1,135 and 1,426 from the A and D subgenomes, respectively; see Materials and Methods). We assigned 2,396 (~94%) SDRGs to nine network modules, and modules 3, 5, and 7 exhibited overall lower expression levels in AT2 (Figure 5A). Notably, there were more D-subgenome genes than A-subgenome genes in these three modules (Figure 5B; 259 *vs*. 100 in Module 3, 128 *vs*. 3 in Module 5, and 59 *vs*. 38 in Module 7; binomial test, *p*-value < 0.05 for all comparisons). Furthermore, the proportion of stably downregulated DEGs from the D subgenome was further enriched in Modules 3 (from 72.14 to 93.07%), 5 (from 97.71 to 100%), and 7 (from 60.82 to 70.59%) (Figures 5B,C; Materials and Methods). The expression patterns of 108 stably downregulated SDRGs in the D subgenome are shown in Figure 5D. They include a number of genes associated with spike development that have been reported previously. For example, *FLOWERING LOCUS T*, a major gene that regulates wheat flowering and yield-related traits (Chen et al., 2014; Liu et al., 2020; Isham et al., 2021), showed increased expression throughout early wheat spike development (Figure 5D). In addition, transcription factor (TF) genes, such as *MYB* and *bHLH* TF genes, exhibited increased expression from early to late development, suggesting that these TFs play important roles in wheat spike development. Twenty-four of 108 SDRGs in the D subgenome that had counterparts in the A subgenome showed bias toward the D genome in Mix in at least one stage but had similar expression levels in AT2 (Supplementary Figure S6). These results imply that downregulation of D-subgenome homoeologs may contribute to the similar spike developmental phenotypes of *T. urartu* and AT2.

On the other hand, the overall subgenome dominance of important A-subgenome SDRGs during spike development may also contribute to the phenotypic similarity of AT2 and *T. urartu* spikes. We further investigated 51 stable A-subgenome HEBs in AT2 (Materials and Methods) and found that the majority of them were intrinsic A-subgenome HEBs (Figure 5E). Intriguingly, spike development-associated transcription factors, including B3, ARF, and MYB factors (Wang et al., 2017; Li et al., 2018), were included in these A-subgenome HEBs, indicating that these TFs tended to be asymmetrically expressed during specific spike developmental stages in the allopolyploid environment. Ubiquitin, F-box protein, and E3 ubiquitin-protein ligase also occurred frequently among the A-subgenome HEB genes, indicating that ubiquitin-related protein processes may play important roles in spike morphology. Moreover, a gene encoding an auxin response factor exhibited A-subgenome HEB (Figure 5E), and this gene has specific roles in the reproductive development of rice, maize, and wheat (Benjamins and Scheres, 2008; Li et al., 2018). These results suggest that some key candidate SDRGs with A-subgenome dominance may also affect spike development in artificially synthesized AT2 wheat.

## Discussion

Studies on multiple plant species have documented many different aspects of allopolyploidy-induced transcriptome shock, including differential gene expression between parents and descendants, homoeolog expression bias (HEB), expression level dominance (ELD), homoeolog-specific expression (HSE), alternative splicing (AS), and expression of non-coding RNAs (Li et al., 2014, 2020; Wang et al., 2016; Ramírez-González et al., 2018; Song et al., 2019; Zhou et al., 2019; Yu et al., 2020). However, there have been few integrative analyses of the changes in gene expression profiles after allopolyploidization at spatial (multiple tissues) and temporal (developmental stages) scales. In the current study, we focused on analysis of DEGs, HEB, and HSE in multiple tissues of a nascent synthetic allotetraploid wheat (AADD) across several developmental stages. We also investigated changes in homoeolog expression connectivity (HEC) in the AT2 allotetraploid using a co-expression network and explored their association with HEB and HSE. A detailed analysis of A- and D-subgenome HEB in important functional homoeologs during AT2 spike development also provided insight into the relationship between spike phenotype and subgenome dominance. Taken together, these analyses provide a useful dataset for tracing allotetraploid-induced expression changes and transcriptomic interactions between two pivotal subgenomes in multiple tissues and developmental stages.

Accumulated evidence has revealed that subgenome expression asymmetry is a common feature of plant allopolyploids and plays a crucial role in the evolutionary success of allopolyploid species (Doyle et al., 2008; Roulin et al., 2013; Yoo et al., 2014; Wang et al., 2016; Li et al., 2020). Nonetheless, the genetic consequences of merging and doubling two “pivotal” wheat genomes are unexplored and worthy of attention. Intriguingly, we found that the two pivotal subgenomes in the synthetic AADD allopolyploid showed immediate and distinct DEGs associated with allopolyploidy-induced transcriptome shock in multiple tissues and developmental stages, and more dramatic responses were detected in the D subgenome. Unlike homozygous alleles in diploid parents faced with external stress, the DEGs of respective homoeologs induced by allopolyploidy (a special type of stress or genomic shock) are supposed to be mutually associated, and their variation degrees of freedom are limited. Major pieces of supporting evidence involve the association of DEGs with HEB in man naturally established allopolyploids, which mainly refers to genome-wide expression skewing toward one of the subgenomes and, therefore, contributed to subgenome dominance (Flagel et al., 2008; Akhunova et al., 2010; Yoo et al., 2013; Li et al., 2014; Zhang et al., 2015; Edger et al., 2017; Powell et al., 2017). Our case demonstrates the applicability of such a concept in synthetic allopolyploid system: overall A-subgenome dominance especially in all five spike developmental stages, which was associated with downregulation of DEGs in the D subgenome (Figure 3B). This result suggests that two pivotal wheat genomes in the same nucleus can accommodate each other and rapidly establish subgenome dominance at both spatial and temporal scales. One technical point should be emphasized here: considering both stable inheritance of preexisting parental expression divergence and independent *de novo* HEB generated after allopolyploidy can result in *prima facie* subgenome expression dominance (Rapp et al., 2009; Chagué et al., 2010; Flagel and Wendel, 2010; Schnable et al., 2011), our conclusion of overall A-subgenome dominance is based on the elimination of inherent expression divergence between diploid parents. Therefore, future transcriptomic investigation of allopolyploid plants should consider the net subgenome expression dominance. Taking our case as an example, the overall A-subgenome expression dominance is observed only when we exclude the intrinsic transcriptomic divergence between the parents (using the *in silico* parental mix as the reference).

Investigation of regulatory divergence by classical allele-specific expression (ASE) and homoeolog-specific expression (HSE) analysis has long been a focus of research on both diploid hybrids and allopolyploids (Wittkopp et al., 2004; Shi et al., 2012; Combes et al., 2015; Bao et al., 2019; Hu and Wendel, 2019; Zhou et al., 2019). However, the regulation divergence of homoeologous expression in allopolyploids, especially within and across multiple tissues and developmental stages, has received less attention. Similar to previous works (Shi et al., 2012; Bao et al., 2019), we confirmed that the overall dominance of *cis*-only regulatory patterns in all tested tissues in synthetic AADD allotetraploid, indicating that the expression regulation patterns of homoeologs tend to be inherited from the divergence of two progenitors by vertical transmission. Consistently, the effect of parental *cis*-element divergence on homoeolog interactions was also seen in the gene co-expression network. All these observations make sense when we consider the long divergence history between *T. urartu* and *Ae. tauschii* (~5.5 MYA) (Glémin et al., 2019) and between far source parents of allopolyploids (Shi et al., 2012), which enabled the accumulation of genetic variations in *cis* elements, such as promoters, enhancers, and gene bodies. Additionally, recent studies in wheat and *Brassica* have suggested subgenome-specific epigenetic modifications are also key factors regulating homoeolog expression bias (Li M. et al., 2021; Wang et al., 2021); however, those latter factors still need further validation in multiple tissues or at different developmental stages in synthesis and natural allopolyploid systems. Notably, despite its prevalence among HSE patterns, the *cis*-only pattern was not particularly conserved among tissues, suggesting that *trans* effects, especially tissue-specific *trans* effects, may balance expression differences between homoeolog mates and play a further fine-tuning role in HSE regulation. In addition, the majority of allopolyploidy-induced rewiring of network modules (~36% homoeologs) was associated with *cis/trans* regulation patterns, and this should be given further attention in future studies. Together, our findings revealed complicated homoeologous regulation *via cis* (major) and *trans* (fine-tuning) effects within different tissues and across developmental stages in initial generations of allopolyploid plants.

Many important agronomic traits in allopolyploid crops are determined by their specific dominant component subgenome(s) (Zohary and Feldman, 1962; Eckardt, 2014; Hao et al., 2019; Cao et al., 2020; Li T. et al., 2021). In allohexaploid common wheat, the dominant A subgenome often determines inflorescence morphology and growth habit, whereas the D subgenome is related to disease resistance and ecological adaptability (Feldman and Levy, 2009). We observed that the spike morphology of our AT2 allotetraploid wheat was similar to that of *T. urartu* (Figure 1C). Interestingly, we also found that SDRGs in the D subgenome were more sensitive to genome merging and doubling, and there were more allopolyploidy-induced DEGs among D-subgenome SDRGs than among A-subgenome SDRGs (Figure 5C). These results suggest that an evolutionarily stable genome, functioning as the major genome, may determine the most important agronomic traits (control of allopolyploid inflorescence morphology by the A genome, in this case), whereas the minor genome with more variable genes may be more sensitive to environmental changes (such as control of disease resistance and ecological adaptability by the D subgenome). In addition, focusing on specific key genes involved in the transition to floral organ differentiation in the wheat inflorescence, most SDRGs were gradually downregulated during spike development. This result implies that the downregulation of cell differentiation genes in the late stage of spike development may be involved in the phenotypic similarity of AT2 to *T. urartu* (Kim et al., 2019). Accordingly, in addition to providing a dynamic overview of subgenome homoeolog interactions, our results also provide insight into fundamental aspects of transcriptional regulation during spike development in allotetraploid wheat. Using other technical advances, such as *in situ* assays, CRISPR-Cas9 genome editing, and proteomic analyses, we will be able to better dissect the molecular regulatory pathways of wheat spike development in future research. Such work should provide valuable candidate genes that may be genetically targeted for yield improvement in wheat.

## Materials and Methods

### Plant Materials and RNA Extraction

The plants used in this study included the newly synthesized allotetraploid wheat (AADD genome, accession AT2) along with its diploid parents, *Triticum urartu* (AA genome, accession TMU38), and *Aegilops tauschii* (DD genome, accession TQ27). Fluorescent *in situ* hybridization (FISH) and genomic *in situ* hybridization (GISH) were conjoined for karyotyping investigation, and only euploid plants were used for further experiments. All the plants were grown under the same controlled growing conditions: 16 h 25°C/8 h 15°C, day/night. We collected samples of different tissues and developmental stages from both AT2 and its diploid progenitors for RNA-seq, which include: shoots and primary roots were collected 4 days after germination; second leaves and mature roots were gathered from trefoil-stage seedlings (Figure 1B); spikes of five successive periods from 0.5 to 2.5 cm in length during stem extension were also obtained (Figure 1C). For each tissue, each of the three biological replicates was pooled from samples collected from three individuals. All collected tissues were immediately frozen in liquid nitrogen. Total RNA was isolated using Trizol (Invitrogen) according to the standard manufacturer's protocol.

### Read Mapping and Expression Quantification

Totally, 81 samples (three genotypes × nine tissues/stages × three replicates) were used for RNA-seq profiling. Library construction and sequencing procedure were performed by standard illumine protocols, and *c*.2.6 billion clean reads (2 × 150 bp) were generated from the Illumina HiSeq2500 (Supplementary Table S1). To estimate and distinguish the accumulated divergence of gene expression (*i*) between two diploid parental species and (*ii*) between parents and AT2, an *in silico* parental mix (hereafter denoted Mix) was constructed by combining the reads of *T. urartu* and *Ae. tauschii* at a ratio of 1:1 for all tissues. For each sample, cleaned RNA-seq reads were mapped against the reference genome constructed by integrated or combined *T.urartu* (http://www.mbkbase.org/Tu/) (Ling et al., 2018) and *A.tauschii* (https://www.ncbi.nlm.nih.gov/bioproject/?term=PRJNA341983) (Luo et al., 2017) genome sequences using HISAT2 (v.2.0.1- beta) (Kim et al., 2015) with default parameters (Supplementary Table S1). Only uniquely mapped paired-end reads were retained for read counting by feature Counts function of the subread package (v1.6.1) (Liao et al., 2014) to generate the count and normalized expression in TPM (transcripts per million reads) values. Only the genes with TPM > 1 among three biological replicates were used for subsequent analysis. To assess a cross mapping rate (a mis-mapping rate) between the two subgenomes for all test tissues, we completed cross mapping validation by aligning both *T. urartu* and *Ae. tauschii* sequencing data to the combined AADD reference genome. We found limited cross mapping rates, which included ~4.9% (4.26–7.51%) of A genome reads mapped to D genome reference and ~0.72% (0.59–1.02%) of D genome reads mapped to A genome reference (Supplementary Table S2). PCA analysis and hierarchical clustering were performed using the prcompfunction and factoextrapackage (http://www.sthda.com/english/rpkgs/factoextra) (Kassambara and Mundt, 2017) in R software (http://www.R-project.org/) (R Core Team, 2013) with default settings.

### Differential Gene Expression Analysis

We examined DEGs (differentially expressed genes) between AT2 and an *in silico* mix using the DESeq2 (Love et al., 2014) package in R software (R Core Team, 2013). Pairwise comparisons of gene expression were made between expression of Mix and AT2 homologs (At *vs*. Ad; Dt *vs*. Dd) at the same developmental stage. Genes with fold change of expression value >2 and FDR adjusted *p*-value < 0.05 were defined as DEGs in each comparison. The DEGs genes in respective genome pair were categorized according to whether the DEG pattern is observed in only one tissue (tissue specific) in 2–7 tissues (intermediate frequency), or more than 8 tissues (constitutive). To identify spike development-related genes (SDRGs), we initially performed pairwise comparisons of gene expression in spike tissues and non-spike tissues to identify genes specifically upregulated in spike tissues in Mix. Specifically, the genes with at least 2-fold upregulation in at least one spike developmental stage were defined as SDRGs. In addition, the genes downregulated in at least three spike stages compared with parents were defined as stably downregulated SDRGs. GO (gene ontology) enrichment analysis of DEGs was performed using the R package clusterProfiler (v3.10.0) (Yu et al., 2012) with the following parameters settings: pvalueCutoff = 0.05, pAdjustMethod = BH, minGSSize = 15, maxGSSize = 500, qvalueCutoff = 0.05.

### Biased Expression Analysis of Homoeologous Gene Pairs

To study the features of homoeolog expression patterns in the allotetraploid AT2, the expression levels of 9,642 *T.urartu-A.tauschii* orthologous gene pairs identified by a python script were monitored. First, CDSs of the *T.urartu* and *A.tauschii* genome were compared against each other by BLASTN (E-value < 1e^−5^). Then, CDS pairs that met following two criteria were considered to be orthologs, which included: (i) aligned regions cover more than 60% of the CDS length for both paired sequences and (ii) aligned regions harbor minimum 90% sequence similarity. The genes with low RNA-seq reads count (both sequences in a homoeologous pair with relative abundance TPM < 1 in all tissues) were discarded.

For each pair of A- and D-subgenome homoeologs, differential homoeolog expression was calculated using DESeq2 to infer homoeolog expression bias (relative contribution of homoeologs to the transcriptome). The homoeologous gene pairs were initially defined as A-biased homoeologous (A-HEB) and D-biased homoeologous (D-HEB) (fold change > 2, FDR adjusted *p*-value < 0.05) and non-biased homoeologous gene pairs (nHEB). Homoeologous gene pairs were further divided into four groups: (*i*) the parental legacy group, in which orthologs and respective homoeologs in Mix and AT2 showed identical HEB in direction; (*ii*) the group displaying allopolyploidy-induced convergent expression, in which HEB occurred in Mix but diminished in AT2; (*iii*) the group of allopolyploidy-induced divergent expression, in which *de novo* HEB was established in AT2, and (*iv*) the group displaying allopolyploidy-induced HEB reversion. In addition, homoeologs exhibiting A-HEB in at least three spike stages were defined as stably A-biased homoeologs. As for net HEB, the approach described by Flagel and Wendel (2010) was used. Briefly, for the 9,621 expressed ortholog/homoeolog pairs, the TPM values of each pair were converted to the ratio of A to D ortholog/homoeolog. These ratios of the allotetraploid were compared to those of parental mix using the Student's *T*-test method (a threshold of *p*-value < 0.05 and fold change >2 was used to assess significance).

### Grouping Homoeologs Based on Their Clustering Modules in Constructed Co-expression Networks

The WGCNA (Langfelder and Horvath, 2008) package in R (R Core Team, 2013) was used to build individual weighted undirected co-expression networks for respective datasets with the *blockwiseModules* function. We input three datasets into the network construction pipeline, including:(*i*) for constructing homoeolog co-expression networks, the TPM RNA-seq expression values of 9,642 homoeologs in Mix and AT2 of 9 tissues/stages were utilized, respectively; (*ii*) for constructing quadruplets co-expression network, expression values of 9,642 homoeologs of Mix and AT2 were used; (*iii*) to build spike-specific network, the TPM values of 2,561 SDRGs in five inflorescence developmental stages of Mix and AT2 were employed. Within the pipeline, the soft thresholding power (β) was set to 18, 18, and 9 for network *i, ii*, and *iii* based on the scale-free topology criterion (Zhang and Horvath, 2005). Groups of closely connected genes, namely modules, were identified by clustering genes based on the topological overlap matrix and cutting the clustering tree into branches by the cutreeDynamic method with parameters: deepSplit = 2, pamRespectsDendro = FALSE, minModuleSize = 30, mergeCutHeight = 0.25. The gene expression pattern within a single module was summarized into a module eigengene (ME), corresponding to the first principal component, which was considered the most representative gene expression in such a module (Fuller et al., 2007).

As for the network *i*, we used the homoeolog expression connectivity (HEC) to assess the degree to which homoeolog pairs diverged in expression. Briefly, the HEC between a pair of homoeologs assesses the degree to which two homoeologs share neighbors in the co-expression network. The higher the connectivity score, the more the homoeologs are assumed to share the same expression profile. According to criteria originally proposed in lotus (Shi et al., 2020), we calculated the hypergeometric-value and the connectivity score, and classified the genes into three groups (Supplementary Figure S4A): Group A with connectivity >0.5 and *P*-value < 0.01, Group B with connectivity 0.5 > × >0.3 and *P*-value <0.01, Group C with connectivity <0.3 and *P*-value >0.99.

### Assignment of *cis-* and *Trans-*Regulatory Divergence

Following the method of analyzing classical allele-specific expression (ASE) (McManus et al., 2010; Bao et al., 2019), we completed homoeolog-specific expression (HSE) analysis to explore the potential regulatory insulation and/or interactions among homoeolog genes in allopolyploid. We identified *cis-* and *trans-*regulatory divergence by using the procedures as described previously (Bao et al., 2019). Specifically, any significant difference between orthologs in Mix was considered as evidence of *cis* and *trans* effects co-regulation [represented by *A*; *A* = log2 (Ad/Dd)], whereas any significant difference between homoeologs in the AT2 was considered evidence of *cis-*regulatory divergence [represented by *B*; *B* = log2 (At/Dt)]; accordingly, *trans* effects were derived by subtracting the expression divergences of gene pairs in AT2 from those of Mix (*A* – *B*). Student's *T*-test was performed to test above three comparisons (comparison *A, B*, and *A* – *B*). All 9,642 expressed homoeologous pairs were specifically classified into seven regulatory categories: (i) *cis* only: *A* = *B, A* ≠ 0, *B* ≠ 0; (ii) *trans* only: *A* ≠ *B, A* ≠ 0, *B* = 0; (iii) *cis* + *trans*: *cis* and *trans* effects of one gene were in the same directions, *A* ≠ *B, A* > 0, *B* > 0 or *A* ≠ *B, A* < 0, *B* < 0; (iv) *cis* × *trans*: *cis* and *trans* effects of one gene were in the opposite directions *A* ≠ *B, A* > 0, *B* < 0 or *A* ≠ *B, A* < 0, *B* > 0; (v) compensatory: *A* ≠ *B, A* = 0, *B* ≠ 0; (vi) conserved: *A* = *B, A* = 0, *B* = 0; (vii) ambiguous: all other patterns of significant tests, with no clear biological interpretation.

## Data Availability Statement

The datasets presented in this study can be found in online repositories. The names of the repository/repositories and accession number(s) can be found below: https://www.ncbi.nlm.nih.gov/, PRJNA807881.

## Author Contributions

LG and BL conceived this project. LG, BL, and XL designed and supervised the project. XM, ZZ, GL, XG, YB, YZ, BW, ML, and TW conducted the experiments and analyzed the data. XM, ZZ, XL, KX, and LG wrote the manuscript. All authors discussed the results and approved the manuscript.

## Funding

This work was supported by the National Natural Science Foundation of China (NSFC #31830006 to BL, #31970238 to LG, and #32100179 to ZZ).

## Conflict of Interest

The authors declare that the research was conducted in the absence of any commercial or financial relationships that could be construed as a potential conflict of interest.

## Publisher's Note

All claims expressed in this article are solely those of the authors and do not necessarily represent those of their affiliated organizations, or those of the publisher, the editors and the reviewers. Any product that may be evaluated in this article, or claim that may be made by its manufacturer, is not guaranteed or endorsed by the publisher.
