# Supplementary material for: Spatial and Temporal Transcriptomic Heredity and Asymmetry in an Artificially Constructed Allotetraploid Wheat (AADD)
Source: Front Plant Sci. 2022 May 16;13:887133. doi: 10.3389/fpls.2022.887133 (PMC9150853; doi:10.3389/fpls.2022.887133)
Supplement: Supplementary file 1 [file Data_Sheet_1.docx]

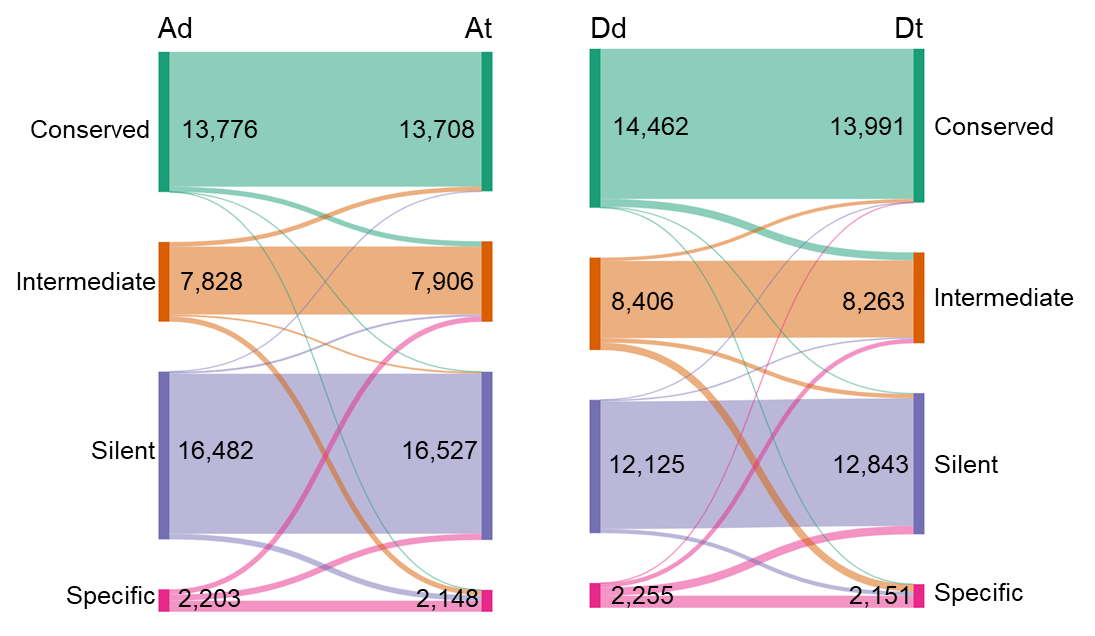


**Supplementary Figure 1.** Changes of expression pattern of the same gene between At and Ad and between Dt and Dd. The number of genes in four gene expression categories:“silent” (without expression in any tissue; 0 tissue), “specific” (TPM > 1 in one tissue), “intermediate” (TPM > 1 in 2 – 7 tissues) and “conserved” (TPM > 1 in 8 – 9 tissues).


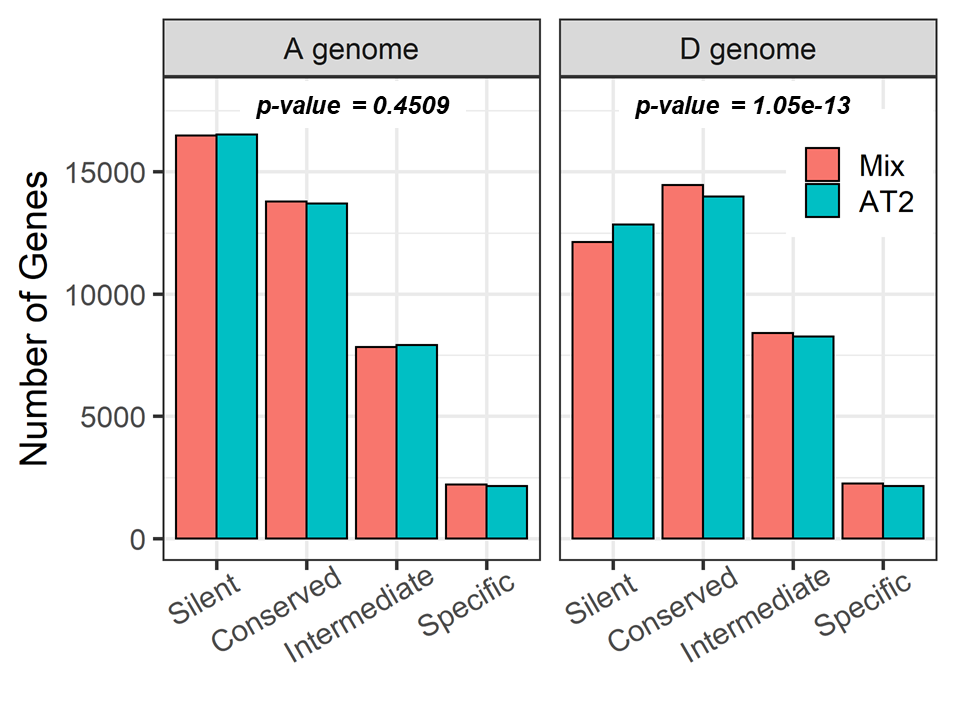


**Supplementary Figure 2.** The numbers of genes that marked as “silent” (without expression in any tissue; 0 tissue), “specific” (TPM > 1 in one tissue), “intermediate” (TPM > 1 in 2 – 7 tissues) and “conserved” (TPM > 1 in 8 – 9 tissues) among genomes/subgenomes in parental mix and AT2 (χ^2^ test).


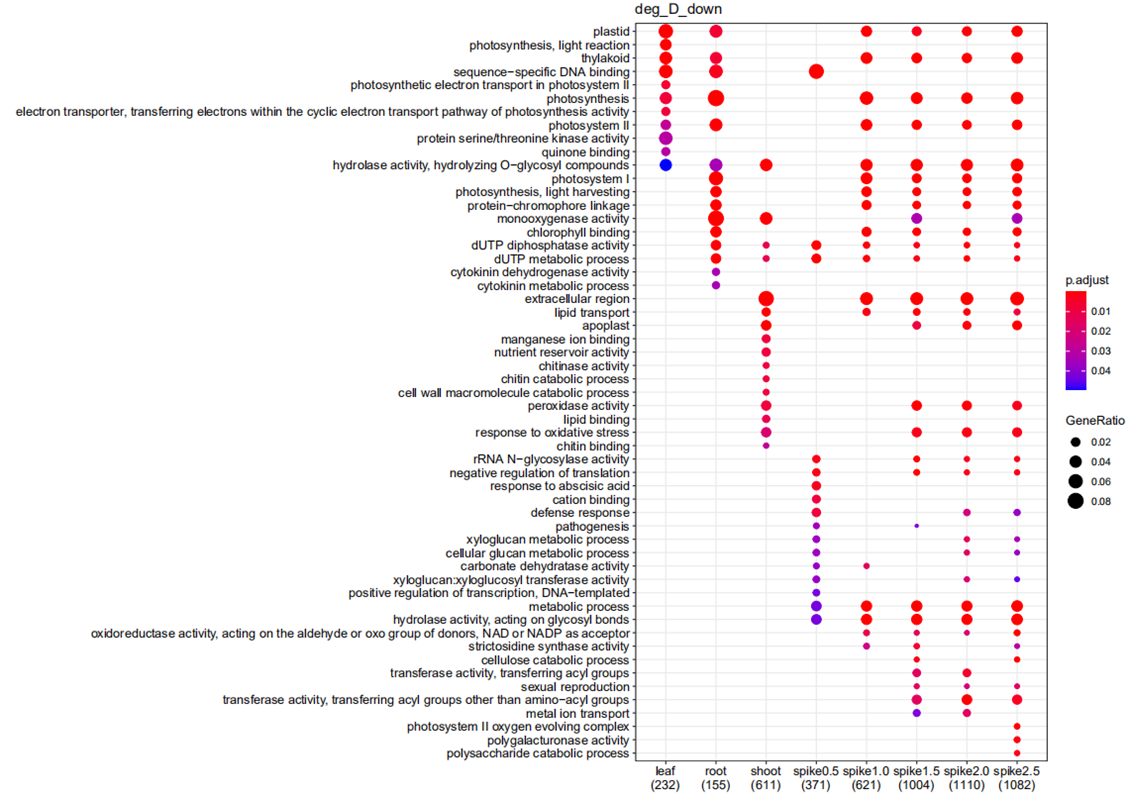


**Supplementary Figure 3.** Enriched GO slim terms of downregulated DEGs from D subgenome across tissues and developmental stages. Abscissa axis represents the count of genes significantly enriched in each term. Node size: gene ratio; node color: P value.


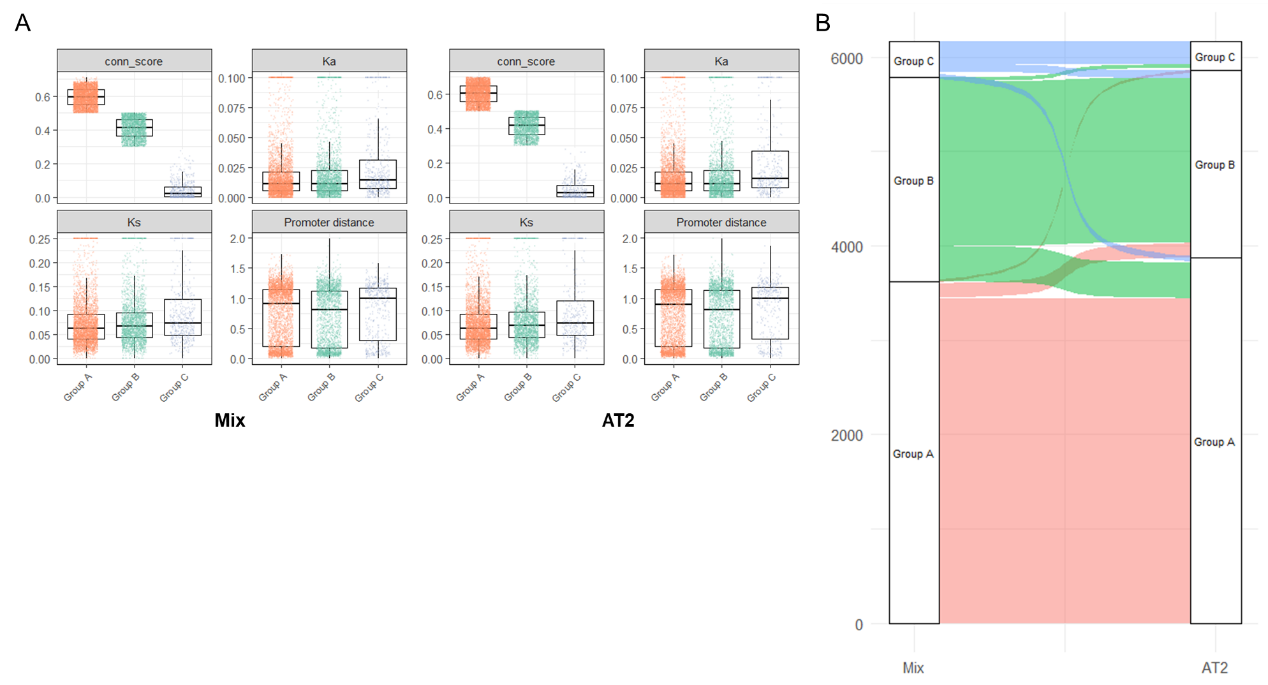


**Supplementary Figure 4.** Changes of homoeolog expression connectivity (HEC) after allopolyploidization. **(A)** Box plots of evolutionary features of homoeologs with different level of expression divergence (group A, group B and group C) in Mix and AT2. Upper left: connectivity score; upper right: Ka (nonsynonymous mutation); lower left: Ks (synonymous mutation); lower right: sequence divergence of promoter region; **(B)** Gene pairs belonging to each of the different groups in Mix (left) and in AT2 (right).


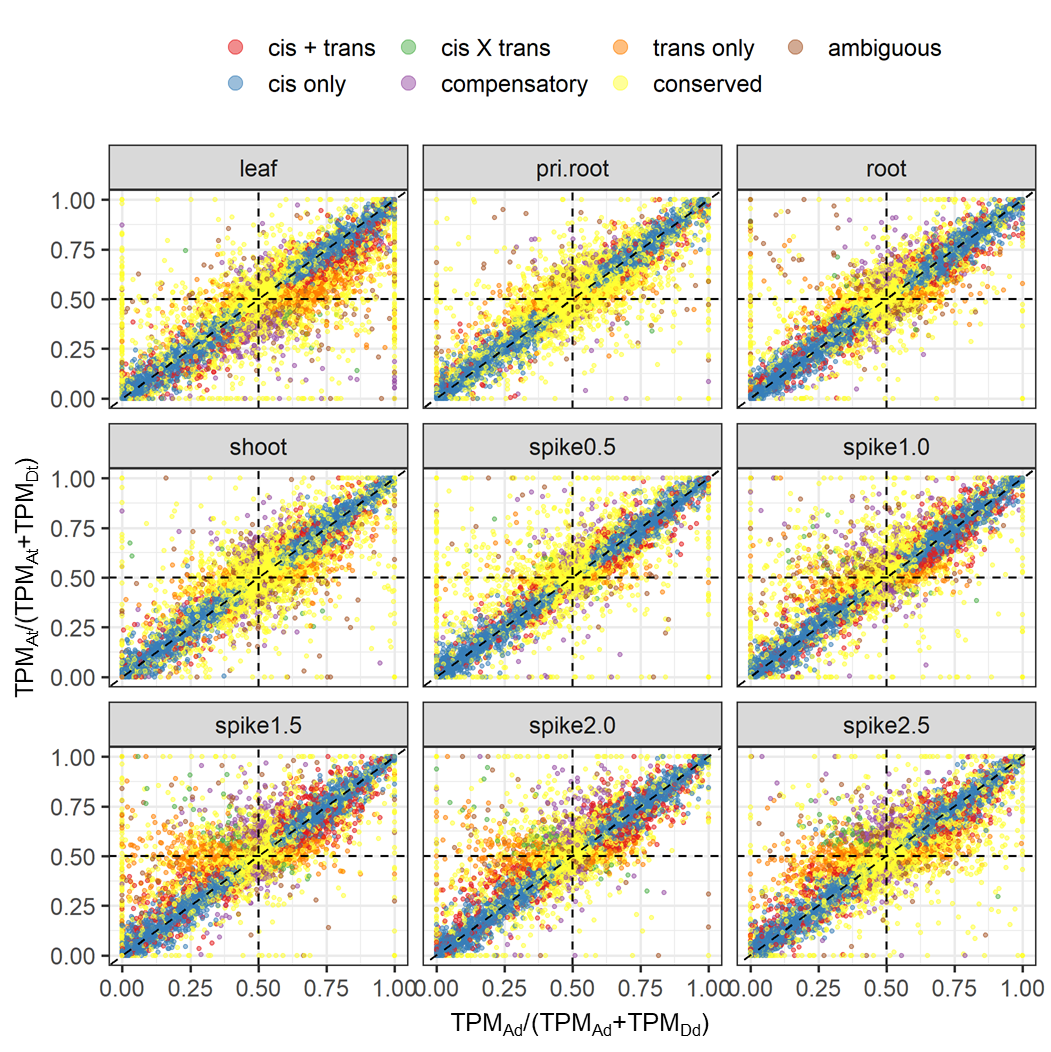


**Supplementary Figure 5.** A scatterplot shows the relationship between the expression ratio of A allele at diploid level (x axis, TPM_Ad_/[TPM_Ad_+TPM_Dd_]) and that of A homoeolog at tetraploid level (y axis, TPM_At_/[TPM_At_+TPM_Dt_]) for genes across all tissues/stages. The colors represent different HSE pattern determined for each gene.


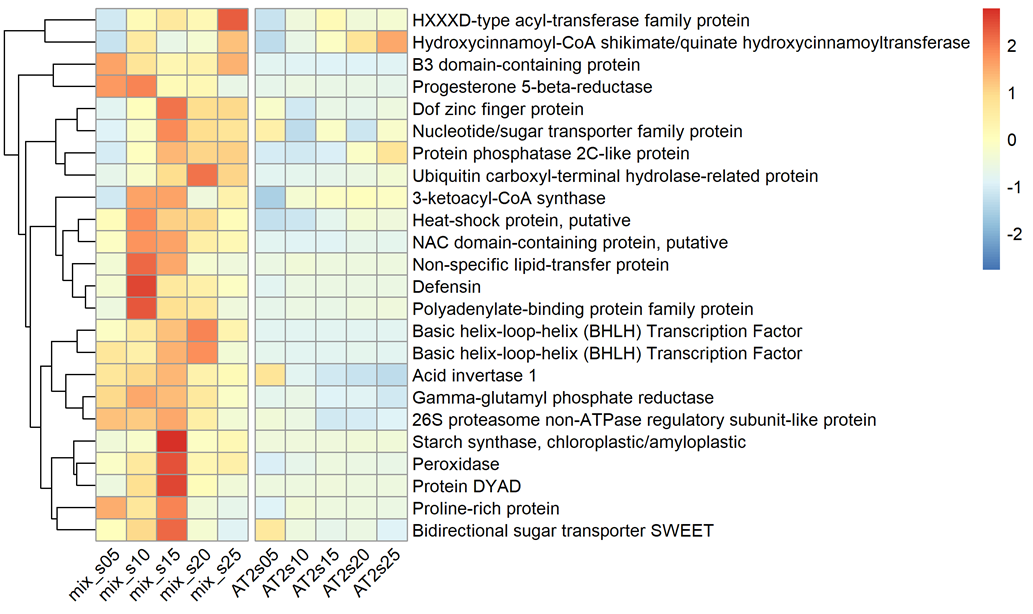


**Supplementary Figure 6.** Expression levels of 24 SDRGs throughout spike development in Mix and AT2. Log2(TPM_D_/TPM_A_) were transformed to Z-scores and are colored blue, white, or yellow to represent low, moderate, or high expression levels.

**Supplementary Table 1.** IIlumina reads obtained from the diploids and tetraploid.

|  | ***T.urartu* (TMU38, AA)** | | | ***A.tauchii* (TQ27, DD)** | | | **AT2 (AADD)** | | |
| --- | --- | --- | --- | --- | --- | --- | --- | --- | --- |
| **Tissue** | **Rep.1** | **Rep.2** | **Rep.3** | **Rep.1** | **Rep.2** | **Rep.3** | **Rep.1** | **Rep.2** | **Rep.3** |
| Leaf | 17,328,000 | 20,556,864 | 19,641,458 | 22,771,264 | 20,451,770 | 19,685,724 | 50,429,952 | 44,714,638 | 38,144,148 |
| Primary root | 19,494,594 | 20,471,094 | 19,649,680 | 23,754,786 | 18,963,486 | 20,145,758 | 45,713,254 | 50,342,164 | 55,330,292 |
| Root | 19,155,458 | 15,033,424 | 16,071,006 | 20,625,276 | 19,950,166 | 19,965,090 | 59,996,548 | 52,612,778 | 54,375,866 |
| Shoot | 17,126,822 | 16,975,416 | 19,470,810 | 20,140,510 | 21,434,186 | 21,155,762 | 54,276,394 | 53,967,274 | 52,208,778 |
| Spike 0.5cm | 20,587,106 | 19,955,944 | 20,227,972 | 19,785,288 | 22,141,516 | 23,147,516 | 49,338,320 | 63,889,948 | 64,220,078 |
| Spike 1.0cm | 20,569,660 | 21,217,608 | 20,143,640 | 18,332,800 | 21,074,794 | 22,555,884 | 55,378,806 | 56,398,096 | 61,335,456 |
| Spike 1.5cm | 15,361,392 | 19,661,376 | 20,840,886 | 23,421,256 | 23,527,990 | 22,582,890 | 60,381,132 | 57,447,770 | 58,785,764 |
| Spike 2.0cm | 20,776,936 | 19,467,202 | 21,847,084 | 23,691,080 | 24,868,572 | 24,105,040 | 58,620,094 | 61,844,480 | 57,216,074 |
| Spike 2.5cm | 21,761,310 | 20,797,856 | 22,715,610 | 24,446,924 | 21,553,120 | 21,030,406 | 57,475,082 | 54,619,488 | 57,055,978 |

**Supplementary Table 2.** Mis-mapping rate of two subgenomes for all test tissues.

|  | **A genome reads mapped to**  **D genome reference (%)** | | | **D genome reads mapped to**  **A genome reference (%)** | | |
| --- | --- | --- | --- | --- | --- | --- |
| **Tissue** | **Rep.1** | **Rep.2** | **Rep.3** | **Rep.1** | **Rep.2** | **Rep.3** |
| Leaf | 7.51 | 7.17 | 7.34 | 0.61 | 0.59 | 0.6 |
| Primary root | 4.75 | 4.8 | 4.84 | 0.62 | 0.65 | 0.66 |
| Root | 5.01 | 5.06 | 5.17 | 0.69 | 0.7 | 0.7 |
| Shoot | 4.75 | 4.7 | 4.93 | 0.65 | 0.65 | 0.75 |
| Spike 0.5cm | 4.41 | 4.26 | 4.33 | 0.68 | 0.82 | 0.73 |
| Spike 1.0cm | 4.51 | 4.46 | 4.49 | 0.77 | 1.02 | 0.75 |
| Spike 1.5cm | 4.4 | 4.6 | 4.52 | 0.78 | 0.78 | 0.81 |
| Spike 2.0cm | 4.51 | 4.45 | 4.31 | 0.76 | 0.75 | 0.75 |
| Spike 2.5cm | 4.28 | 4.3 | 4.5 | 0.77 | 0.77 | 0.78 |

**Supplementary Table 3.** Expression pattern of homoeologous gene pairs in *in silico* mix and synthetic tetraploid wheat (AT2).

| **Groups** | **Categories** | **leaf** | **pri.root** | **root** | **shoot** | **spike0.5** | **spike1.0** | **spike1.5** | **spike2.0** | **spike2.5** |
| --- | --- | --- | --- | --- | --- | --- | --- | --- | --- | --- |
| (i) Parental legacy | A-HEB_A-HEB | 813  (8.84) | 649  (6.82) | 779  (8.81) | 688  (7.2) | 800  (8.47) | 734  (7.76) | 713  (7.51) | 765  (8.06) | 695  (7.32) |
|  |  |  |  |  |  |  |  |  |  |  |
|  | D-HEB_D-HEB | 1257  (13.67) | 1300  (13.66) | 1399  (14.69) | 1373  (14.36) | 1385  (14.66) | 1412  (14.92) | 1386  (14.59) | 1406  (14.82) | 1347  (14.19) |
|  |  |  |  |  |  |  |  |  |  |  |
|  | nHEB_nHEB | 6286  (68.36) | 6989  (73.44) | 6726  (70.63) | 6818  (71.3) | 6698  (70.88) | 6677  (70.57) | 6557  (69.03) | 6563  (69.18) | 6704  (70.61) |
|  |  |  |  |  |  |  |  |  |  |  |
| (ii) Polyploidy induced convergence | A-HEB_nHEB | 289  (3.14) | 160  (1.68) | 139  (1.46) | 200  (2.09) | 98  (1.04) | 115  (1.22) | 238  (2.51) | 153  (1.61) | 227  (2.39) |
|  | D-HEB_nHEB | 190  (2.07) | 160  (1.68) | 112  (1.18) | 148  (1.55) | 103  (1.09) | 144  (1.52) | 306  (3.22) | 243  (2.56) | 242  (2.55) |
| (iii) Polyploidy induced divergence | nHEB_A-HEB | 190  (2.07) | 132  (1.39) | 220  (2.31) | 194  (2.03) | 217  (2.3) | 236  (2.49) | 179  (1.88) | 224  (2.36) | 163  (1.72) |
|  | nHEB_D-HEB | 171  (1.68) | 126  (1.32) | 148  (1.55) | 140  (1.46) | 148  (1.57) | 144  (1.52) | 119  (1.25) | 133  (1.4) | 116  (1.22) |
| (iv) Polyploidy induced HEB reversion | A-HEB_D-HEB | 0 | 0 | 0 | 0 | 1 | 0 | 1 | 0 | 1 |
|  | D-HEB_A-HEB | 0 | 0 | 0 | 1 | 0 | 0 | 0 | 0 | 0 |
|  |  |  |  |  |  |  |  |  |  |  |
